# Biofuser: a multi-source data fusion platform for fusing the data of fermentation process devices

**DOI:** 10.3389/fdgth.2024.1390622

**Published:** 2024-10-21

**Authors:** Dequan Zhang, Wei Jiang, Jincheng Lou, Xuanzhou Han, Jianye Xia

**Affiliations:** ^1^ State Key Laboratory of Bioreactor Engineering, East China University of Science and Technology, Shanghai, China; ^2^ Engineering Biology for Biomanufacturing, Tianjin Institute of Industrial Biotechnology, Chinese Academy of Sciences, Tianjin, China

**Keywords:** bioprocess optimization, multi-source heterogeneous data, multi-source data fusion, Biofuser, intelligent biomanufacturing

## Abstract

In the past decade, the progress of traditional bioprocess optimization technique has lagged far behind the rapid development of synthetic biology, which has hindered the industrialization process of synthetic biology achievements. Recently, more and more advanced equipment and sensors have been applied for bioprocess online inspection to improve the understanding and optimization efficiency of the process. This has resulted in large amounts of process data from various sources with different communication protocols and data formats, requiring the development of techniques for integration and fusion of these heterogeneous data. Here we describe a multi-source fusion platform (Biofuser) that is designed to collect and process multi-source heterogeneous data. Biofuser integrates various data to a unique format that facilitates data visualization, further analysis, model construction, and automatic process control. Moreover, Biofuser also provides additional APIs that support machine learning or deep learning using the integrated data. We illustrate the application of Biofuser with a case study on riboflavin fermentation process development, demonstrating its ability in device faulty identification, critical process factor identification, and bioprocess prediction. Biofuser has the potential to significantly enhance the development of fermentation optimization techniques and is expected to become an important infrastructure for artificial intelligent integration into bioprocess optimization, thereby promoting the development of intelligent biomanufacturing.

## 1 Introduction

Bioprocess optimization is a critical technology in the field of microbiology and, in conjunction with synthetic biology, serves to validate the capacity of high-yield strains constructed via synthetic biology, providing crucial direction for further refinement of such strains. When integrated with industrial production, bioprocess optimization has been shown to lead to significant increases in product output, as well as reductions in production costs. Recent years have witnessed substantial progress in synthetic biology, with the introduction and refinement of high-throughput technology resulting in improved yields of high-yield strains (1). At the same time, bioprocess optimization has also advanced with the use of high-throughput parallel bioreactors (2–5), which have facilitated reductions in the time and cost required for biological process optimization. The use of off-gas mass spectrometry has enabled more precise acquisition of off-gas data (6), while the availability and accumulation of various process measurement instruments and sensors have broadened the range of process parameters (5, 7, 8). However, the heterogeneity of multi-source data poses significant challenges for biological process researchers in terms of data processing and represents a considerable obstacle to the automation and intelligence of biological processes (9).

The challenge of processing multi-source heterogeneous data in biological processes arises from differences in data collection methods, data formats, and data presentation across sources. Effective methods for data acquisition, preprocessing, and fusion are essential to address this challenge. These processes demand the comprehensive application of knowledge from various disciplines, including computer science, biology, chemistry, chemical engineering, and related fields. Several tools (see Table 1) have been developed to address these difficulties, such as the Inventory of Composable Elements (ICE) (10), which is used to manage seed information, replacing the inefficient recording methods and improving update and search efficiency. The Experiment Data Depot (EDD) (11) is used for the recording of experimental data and metadata, aggregating data from devices and manual inspection through various protocols, enabling automatic data collection and visualization. The Automated Recommendation Tool (ART) (12, 13) can provide model prediction and experimental recommendation for synthetic biology, combining biology and machine learning to promote the development of synthetic biology. These tools provide valuable support for the development of synthetic biology (14) and are powerful examples of cross-disciplinary research. However, these tools are not specifically designed for bioprocess optimization. Moreover, they are nothing more than simple combination of data from devices and lack the fusion data used in biological process analysis. Therefore, there is a need for the development of specialized tools and platforms for bioprocess optimization that can effectively integrate multi-source heterogeneous data and facilitate intelligent biomanufacturing.

**Table 1:** Comparison of tools for data management and fusion in biological processes.

Tool	Field	Data source	Data collection	Data preprocessing	Data fusion	Key advantage	References
ICE	Seed management	Simple	Manual	None	None	Efficient recording and updating of seed information	(10)
EDD	Experimental data recording	Simple	Auto, manual	None	None	Automatically aggregates experimental data from different protocols	(11)
ART	Model prediction in synthetic biology	Simple	Auto, manual	None	Simple data combination	Provides predictive models and experimental recommendations for synthetic biology	(12)
Biofuser	Fermentation process optimization	Complex	Auto, manual	Filtering, filling, normalization	Multi-source data fusion	Real-time fusion of multi-source heterogeneous data for fermentation processes	This study

Multi-source data fusion has emerged as a powerful approach for analyzing complex biological systems by integrating diverse types of data to provide a more comprehensive understanding of biological processes. Over the years, a range of technical approaches have been developed to address the challenges of combining data from multiple sources, including network-based methods (15), machine learning methods (16), matrix factorization methods (17), deep learning methods (18), and integrated methods (19). These methods have demonstrated varying levels of success in a range of applications, from drug discovery to cancer research. Despite their promise, however, each method has its own strengths and limitations, and the development of new approaches that can better leverage the strengths of each method while overcoming their limitations is a critical need in the field of biological process research. In this context, it is important to identify the key challenges and opportunities for advancing the field of multi-source data fusion in biological process development. One key challenge is the development of methods for integrating data from diverse sources, including transcriptomic, proteomic, and metabolomic data, as well as data from sensors and other monitoring devices. This requires the development of new algorithms and computational tools that can effectively process and integrate these different types of data. Another challenge is the need for more robust methods for data preprocessing, quality control, and normalization to ensure that the resulting integrated data are accurate and reliable. These methods must be able to handle missing data, outliers, and other sources of noise and must be scalable to handle large datasets. Finally, there is a need for more sophisticated methods for data analysis and interpretation, including the development of new machine learning algorithms and other computational tools that can identify patterns and relationships in the integrated data and provide insights into the underlying biological processes.

**Figure 1:**
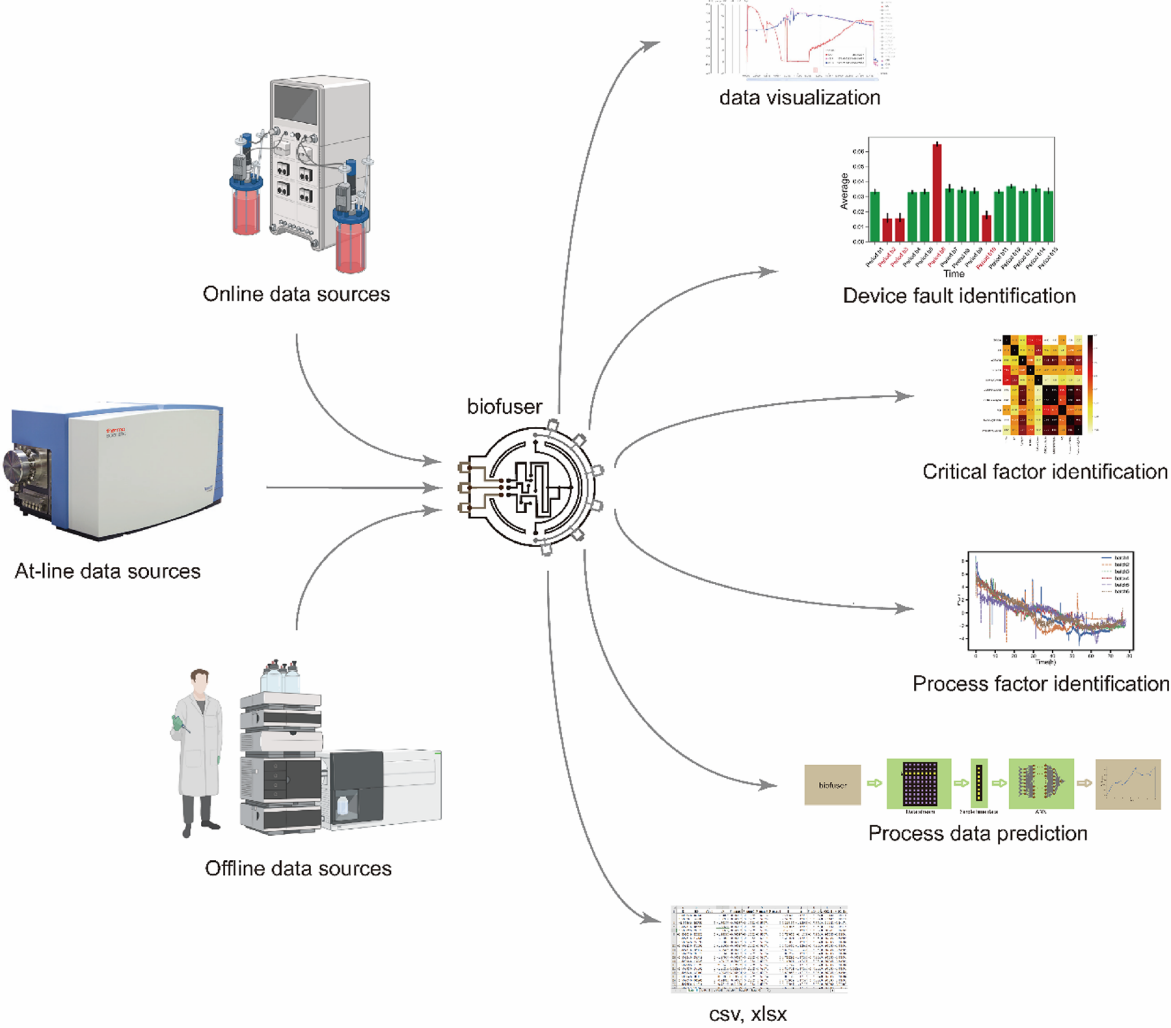
Overview and crucial capabilities of Biofuser. Biofuser collects data from different data sources, stores them into database, and is capable of data visualization, device faulty identification, critical factor identification, process factor identification, process data prediction, and downloading data for other analysis techniques.

**Figure 2:**
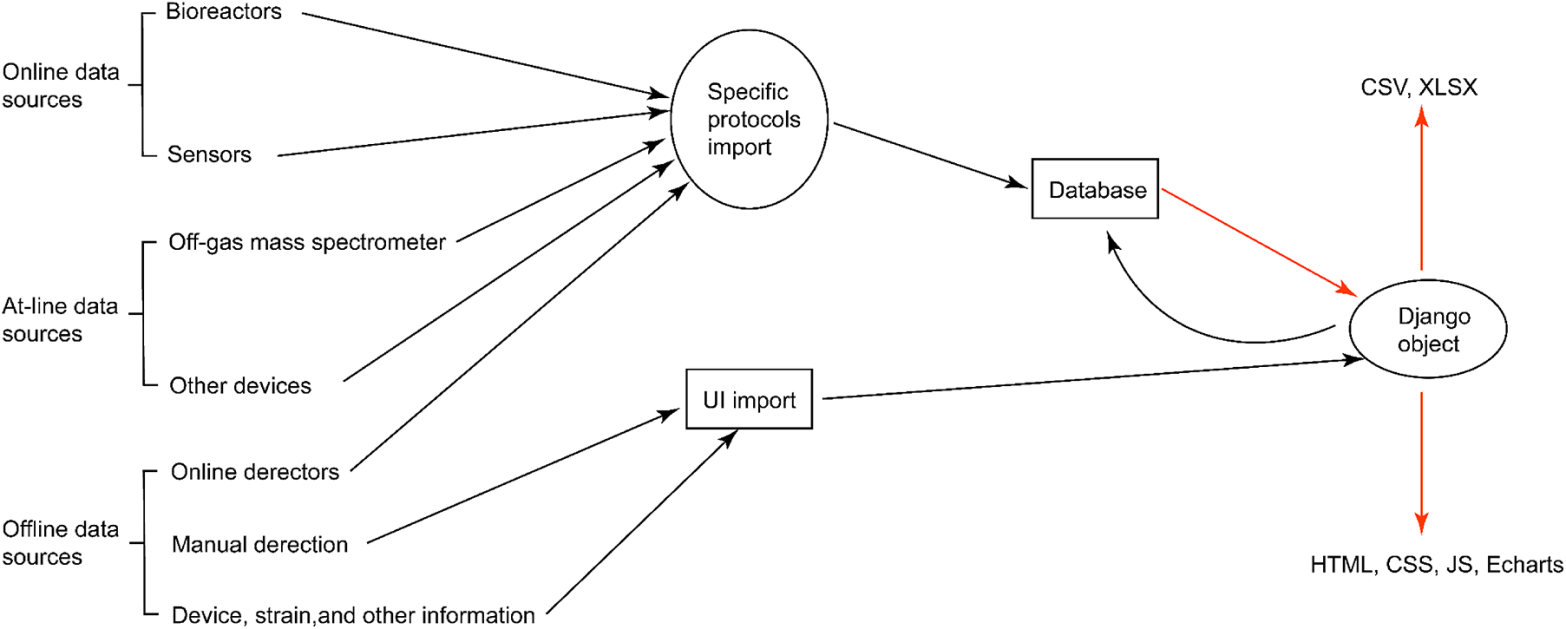
Modules of Biofuser for source data collection and output. A series of simultaneous experiments will be described in Biofuser as an example. There are two types of data collection: communication protocol based automatic import and manual import. Protocol based import method import data directly into the database by the upper computer in the laboratory depending on the data generating devices’ communication protocols, such as OPC, MQTT, and Modbus. Manual import is implemented through a user interface inputting field which allow the system user input data.

In this work, we propose Biofuser (see Figure 1), a multi-source fusion platform that has been designed for the real-time collection, preprocessing, and fusion of fermentation process data. The platform collects data that is categorized as online data, at-line data, and offline data mainly from auto-collection through specific communication protocols or from manual input through user interface (see Figure 2). The first method employs computer communication technologies, such as Modbus protocol, Object Linking and Embedding for Process Control protocol (OPC), Message Queuing Telemetry Transport protocol (MQTT), while the second method involves manual input. The collected data undergoes preprocessing, which includes data filtering, mean filling, interpolation filling, zero filling, and data normalization based on the unique characteristics of each data source. Biofuser applies biological fermentation experience and theory to the data from different sources to generate fused parameters. Unlike EDD, Biofuser has been specifically designed for the fermentation process, storing the original data from the equipment and displaying the fused data on the front-end. By leveraging its specialized design, Biofuser can more effectively integrate and fuse multi-source heterogeneous data, providing valuable insights into the fermentation process and supporting bioprocess optimization.

## 2 Methods

### 2.1 Core components of Biofuser

Biofuser is composed of two components, namely real architecture components and logical architecture components. The former includes devices, communication protocols, database, Django, model, view, and template. The real architecture component is responsible for collecting and storing data from various sources, managing communication protocols, and providing a user interface for data visualization and analysis. The latter includes data acquisition, data preprocessing, and data fusion. It is responsible for processing the collected data, ensuring data quality and consistency, and integrating the data from multiple sources into a uniform format. By combining these two components, Biofuser can effectively integrate multi-source heterogeneous data and provide valuable insights into the fermentation process optimization.

**Figure 3:**
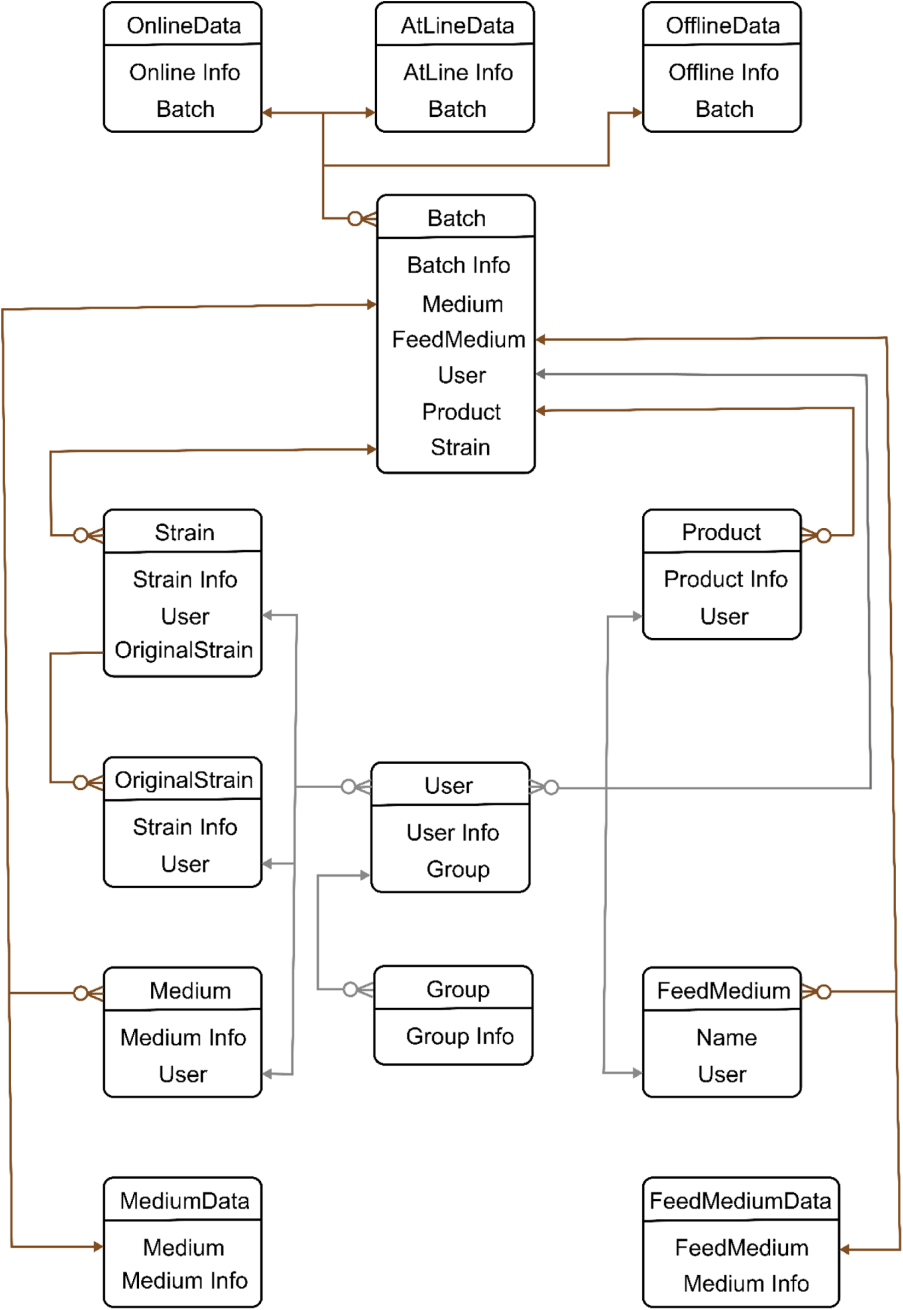
Entity relationship (ER) diagram of Biofuser background database. ER diagram has two relationship lines: user line and data line. User line is designed to set data access permissions. Different users have different permissions such as viewing, editing, exporting, and importing. Centered on batch, data line is designed to series all data before and after fermentation.

• Data sources: Data sources can be categorized as online, at-line, and offline based on their collection method and characteristics. Online data sources are continuously collected with a high frequency, such as data from sensors installed in a bioreactor. At-line data sources require special processing and have an irregular collection frequency. Examples of at-line data sources include off-gas mass spectrometry, which measure the composition of off-gas and it always not aligned with the corresponding online data of the same process. Offline data sources require manual processing or inspection, such as samples taken from the bioreactor at different time points for analysis in a laboratory.
• Communication protocols: Compatibility with the data source's supported protocol, including common industrial protocols such as OPC and Modbus, which is crucial for effective data acquisition.
• Database: A relational database has been designed (ER diagram of the data base is shown in Figure 3) to store data acquired from different sources as well as user-defined data. This database provides data support for the Django server.
• Django: Django is a web development framework built on the Python programming language. It is structured around three principal components, which form the main framework: the model component, view component, and template component.
• Model component: It represents the data structure of the application, defining the fields and relationships between the data. It is responsible for handling data storage, retrieval, and manipulation, and provides an interface for accessing the data from the view and template components.
• View component: The view component handles the logic of the application, processing requests from the user and returning appropriate responses. It interacts with the model component to retrieve and manipulate data, and with the template component to generate the appropriate HTML pages to be displayed to the user.
• Template component: The template component is responsible for the presentation of the application, defining the layout and design of the user interface. It interacts with the view component to access the data to be displayed, and generates the appropriate HTML code to be rendered in the user's web browser.

### 2.2 Key function of Biofuser

User and experimental metadata are crucial components of a bioprocess management system. Proper management of user and experimental metadata can help ensure data security and integrity, and enable efficient collaboration and decision-making in bioprocess development and optimization.

#### 2.2.1 User management

**Figure 4:**
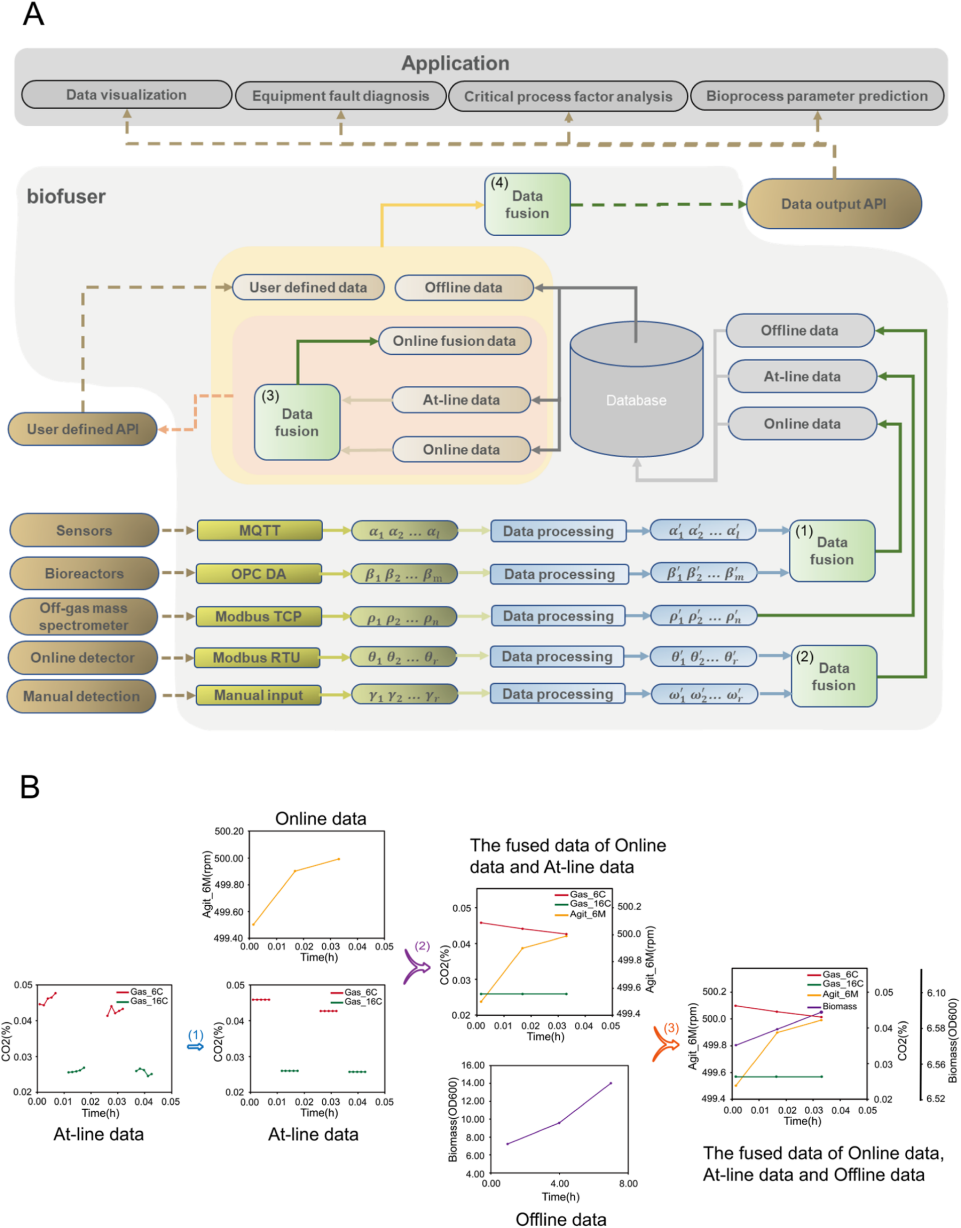
Key structure and functions of Biofuser. **(A)** Overall diagram for the structure of Biofuser. Initially, Biofuser collects data from diverse data sources by means of different communication protocols. Subsequently, the collected data is fused based on the characteristics of the devices, protocols, and data itself. Finally, the processed data is categorized into online data, at-line data, and offline data, and stored in a relational database. Finally, Biofuser retrieves data from a relational database and performs in-depth data fusion to integrate the collected data into a cohesive dataset, which is subsequently transmitted to the application end. **(B)** Illustration the main fusion of different data source. (1) Do averaging and replacing for off-gas measurement of each measuring channel (At-line data). (2) Do aligning of data from online bioreactor primary variables (Online data) and off-gas spectrometry variables (At-line data). (3) Do interpolating of Offline data to fuse it with aligned Online and At-line data.

User management in a bioprocess management system is an important aspect of ensuring data security and integrity. The system should allow users to register and fill in their basic information, account, and password, which should be encrypted using secure algorithms like Message-Digest Algorithm 5 (MD5). Only authorized users should have access to the system, and the account and password should be the only credentials required for login. Additionally, to enable sharing of user information between projects, a grouping function should be set up (see Figure 4), allowing users in the same group to view the group accessed data. To prevent data leakage, the users in the same group are divided into responsible and ordinary users, and only responsible users have the access for downloading data. Ordinary users do not have the ability to delete their accounts and only administrators have permission to delete a user account.

#### 2.2.2 Experimental management

Experimental management in the bioprocess management system is designed according to batches (see Figure 3). At batch experiment design stage, metadata of the design like strain information, medium composition, operation conditions, control strategy were organized as an individual table in the database. Users that create the batch experiment have access to the corresponding experiment data, and the connectivity was stored in the experiment data.

#### 2.2.3 Data input

There are two input modes for data entry (see Figure 2) in Biofuser, which are designed to accommodate different types of data. The first input mode is for data that can be imported directly from the device, specific protocols are used for data import. The second input mode is for data that cannot be directly imported by the device, such as strain information, medium composition, manual measurement data, etc. This type of data was imported through a user interface designed by Django.

#### 2.2.4 Data fusion

Biofuser employs three distinct methods for data fusion (see Figure 4). The first method involves the integration of communication protocols between different data sources through the use of synchronous acquisition or asynchronous processing, allowing for classification of the fused data as Online, At-line, or Offline data. The second method utilizes the nearest time alignment and Lagrange linear interpolation filling techniques to fuse data from different time intervals. The third method aligns offline data with online data through first aligning the former to the corresponding time of the latter and then applying interpolation filling. These data fusion techniques enable the integration of data from diverse sources and lead to the computation of novel parameters via the fusion process. For example, data from the off-gas mass spectrometer and bioreactors are merged into Oxygen Uptake Rate (CER), Carbon-dioxide Escape Rate (OUR), and Respiratory Quotient (RQ).

#### 2.2.5 Data output

**Figure 5:**
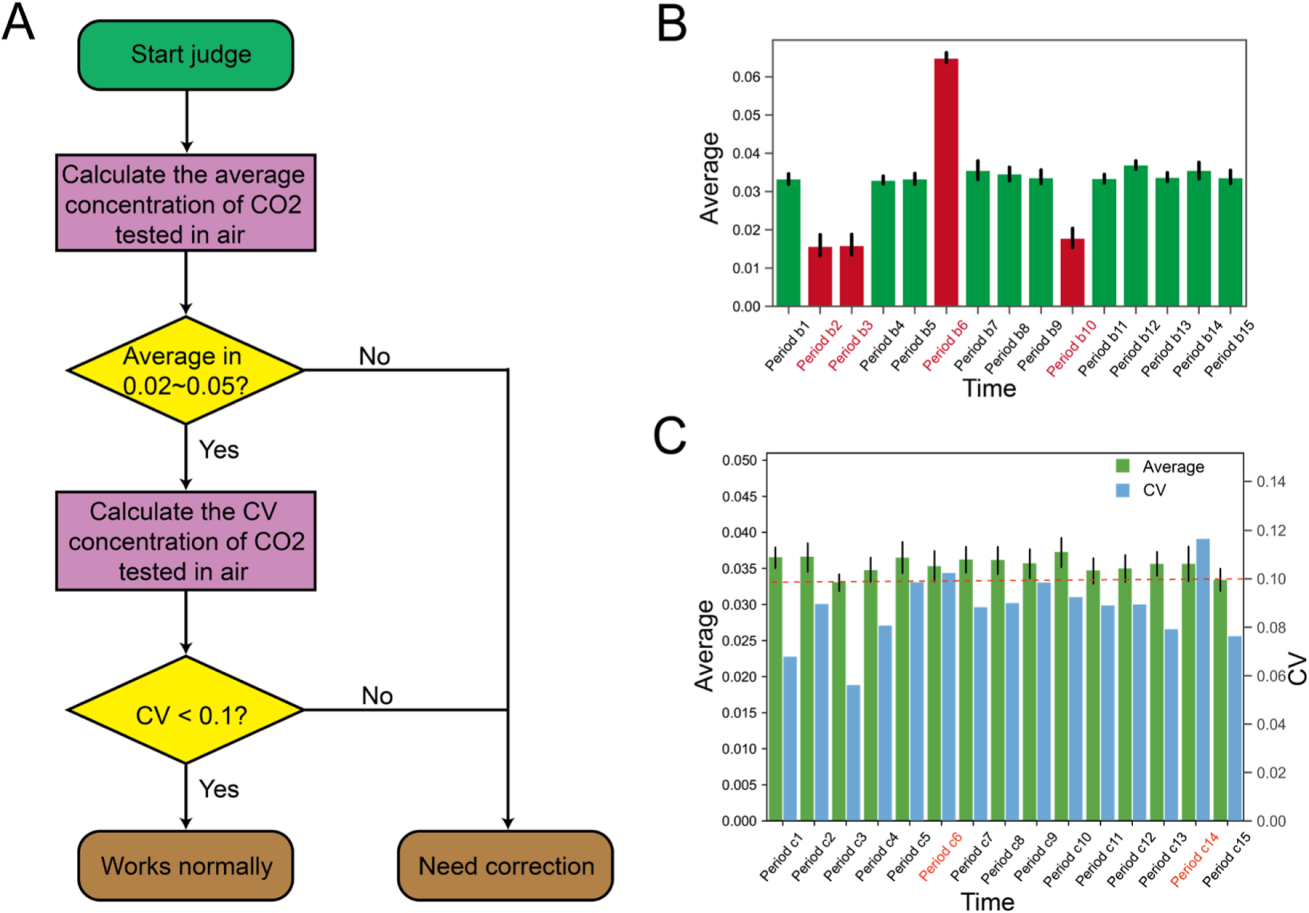
Device faulty identification. **(A)** The process of diagnosing abnormalities in the off-gas mass spectrometer. Initially, the off-gas mass spectrometer data collected by Biofuser is utilized to compute the mean CO_2_ concentration in the air. Subsequently, the coefficient of variation is determined based on the aforementioned mean value. If all the predetermined criteria are met, the tail gas mass spectrometer is deemed to work normally; otherwise, it is deemed to require correction. **(B)** Device faulty identification based on the average value. Two threshold values, namely 0.02 and 0.05, are established for the mean CO_2_ concentration in the air detected by the tail gas mass spectrometer. Any detected mean CO_2_ concentration that is above 0.05 or below 0.02 is considered an anomaly in the equipment. **(C)** Device faulty identification based on CV. The coefficient of variation is evaluated using the CO_2_ concentration data obtained from the air samples collected by the tail gas mass spectrometer. If the coefficient of variation calculated over a specified time interval exceeds 0.1, the equipment is classified as anomalous.

**Figure 6:**
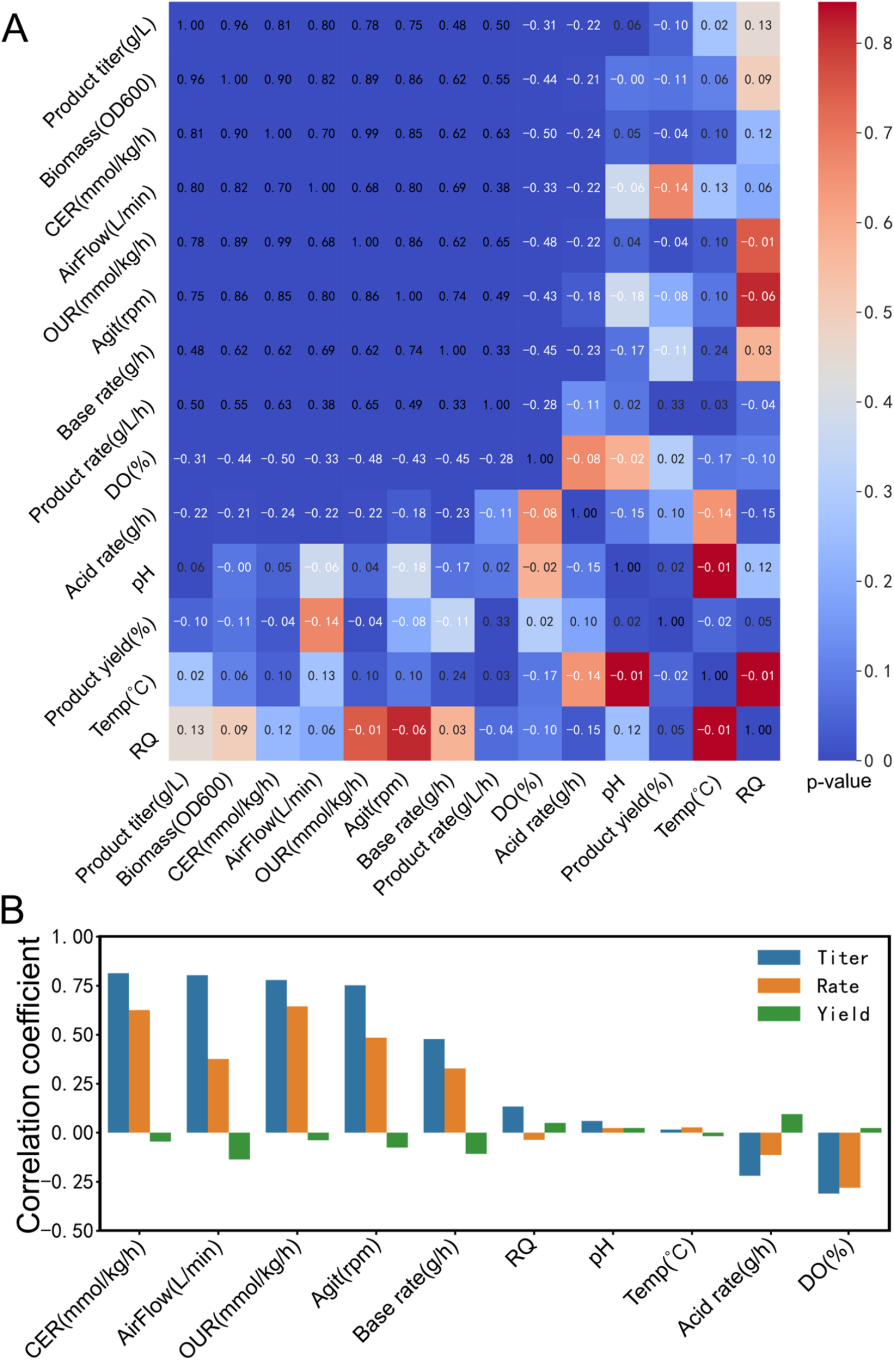
Spearman correlation analysis to identify critical process factors. **(A)** Correlation analysis of fermentation process factors and product TRY. Different colors represent the *p*-value between titer, rate, and yield with other bioprocess parameters, including CER, air flow rate, OUR, agitation speed, base flow rate, RQ, pH, temperature, acid flow rate, and dissolved oxygen. The numerical values in the figure represent the correlation coefficients between these parameters, with white font indicating negative correlation and black font indicating positive correlation. **(B)** Spearman correlation coefficient of fermentation process factors and product TRY. The color-coded bars represent the Spearman correlation coefficients between titer, rate, and yield, and various fermentation process parameters.

**Figure 7:**
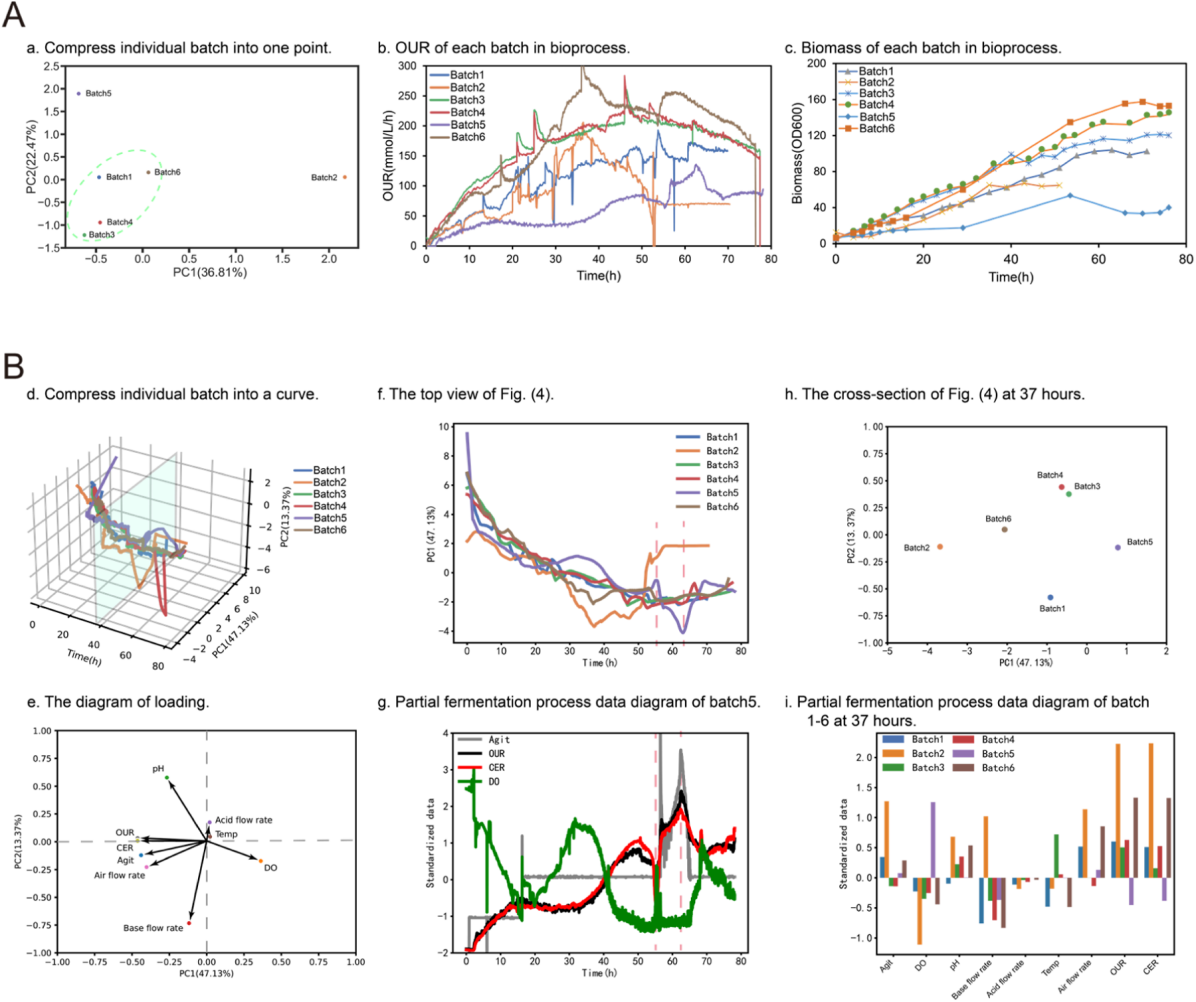
Principal component analysis for bioprocess factor analysis. **(A)** Shows the first PCA method for processing fermentation process data. Plot **(a)** shows the scoring diagram of PCA (PC1 and PC2). The data from each batch were flattened into one row and concatenated to form a new matrix. Then, PCA was applied to reduce the dimensionality of the data to two dimensions (PC1 and PC2). Finally, the scores plot was generated using the reduced matrix. The scores plot is a visual representation of the similarity between different samples based on their PCA scores. Plot **(b)** and **(c)** present the OUR and biomass of each batch, respectively. **(B)** Shows the second PCA method for processing fermentation process data. Plot **(d)** shows the PCA scores (PC1, PC2, and time) at each time point. The multi-batch data was reduced to two dimensions (PC1 and PC2) through dimensionality reduction. Then, a Savitzky-Golay filter was utilized for smoothing. Finally, the smoothed data was combined with time to form a three-dimensional score plot. Plot **(e)** shows the loading data after PCA dimensionality reduction. Plot **(f)** and **(g)** present the top view and the fermentation process data plot of batch 5 for plot **(d)**, respectively. Plot **(h)** and plot **(i)** depict the sectional plot of plot **(d)** at 37 h and the fermentation process data plot of each batch, respectively.

**Figure 8:**
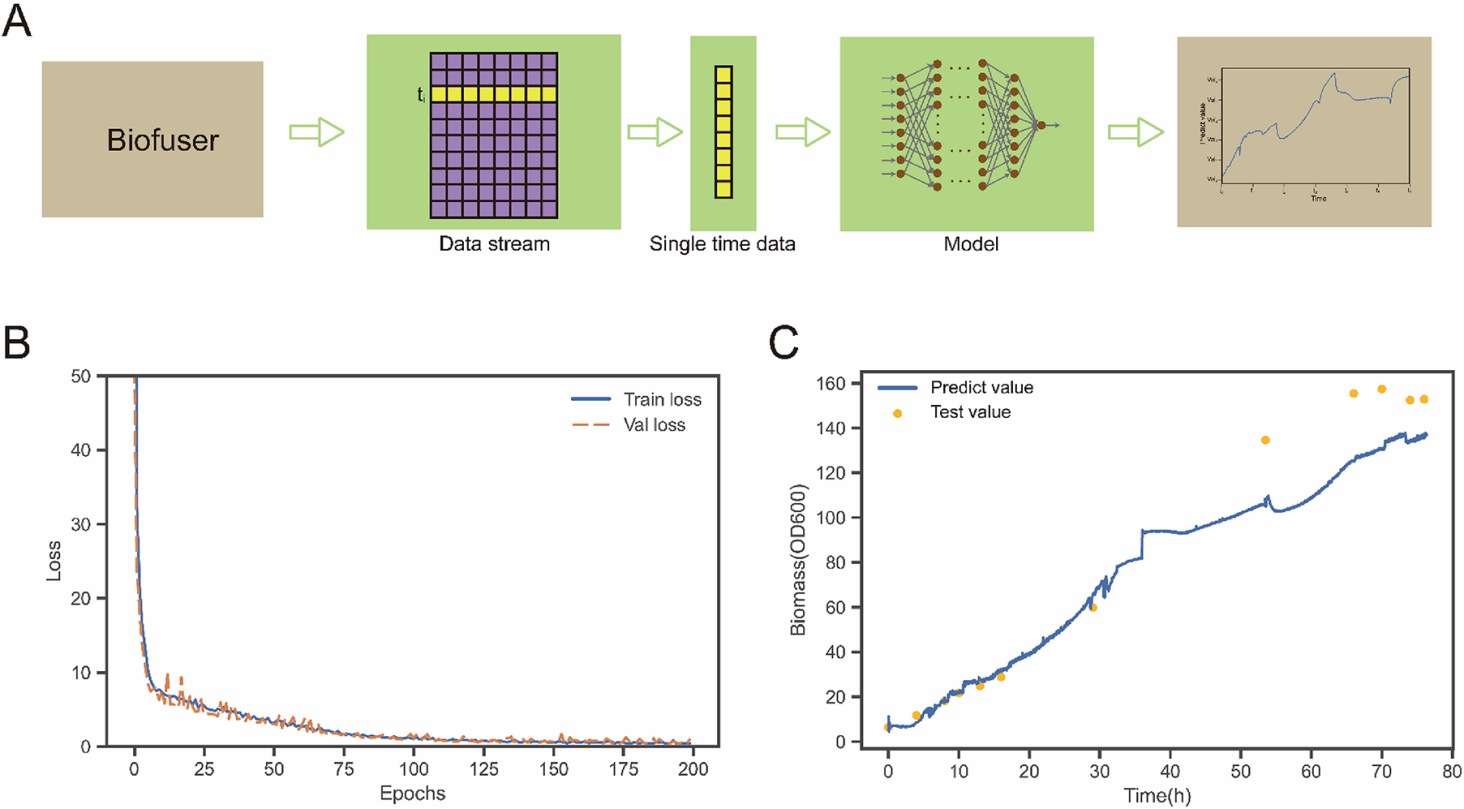
Bioprocess data prediction. **(A)** Is a schematic diagram of the connection process between Biofuser and ANN. The output data stream of Biofuser is processed into the input data of ANN, and the corresponding biological process parameters are output through the ANN model. **(B)** Shows the loss function diagram of ANN model in the case. The loss function of the training set and the verification set converge. **(C)** Shows predicted and test values of a batch of experimental data. In the early stage of fermentation, the predicted value was similar to test values. At the later stage of fermentation, predicted values were 11.0%–19.4% lower than test values.

Biofuser transmits data through a particular Application Program Interface (API). The operation data of the device is transmitted out for device faulty identification (see Figure 5). The fused data were sent out in batches for critical factor analysis (see Figure 6) and biological process factor analysis (see Figure 7). The fused data is sent out in the form of data stream for biological process data prediction (see Figure 8). In addition, Biofuser can export data to CSV and XLSX formats.

## 3 Results and discussion

### 3.1 Construction methodology of Biofuser

Biofuser is a bioprocess management system that focus on automating the collection, preprocessing, and fusion of fermentation process data obtained from laboratory devices. By automating these processes, Biofuser significantly reduces the amount of time required for manual processing and prevents human calculation errors, resulting in more accurate and reliable data. In addition, Biofuser stores data in a structured manner, which facilitates future intelligent development. By leveraging machine learning and other advanced analytics techniques, the system can provide valuable insights into the fermentation process.

In the following section, we will provide an overview of the construction methodology of the Biofuser. As illustrated in Figure 4, Biofuser primarily consists of two main parts. Firstly, the platform collects data from the laboratory equipment and stores it in the database. Subsequently, it retrieves the stored data from the database, processes it, and transmits the data to the user interface for visualization or further data analysis.

#### 3.1.1 Data transmission from devices to database

Biofuser is designed to be able to communicate with as more bioprocess instruments as possible, currently, bioreactors (Bioflo & Celligen 310 and Bioflo & Celligen 115, New Brunswick Scientific, USA, providing primary online variables, like pH, DO, temperature, impeller rotation speed, air flow rate, etc. of the bioprocess), off-gas mass spectrometry (MAX300-LG, Extrel, USA, providing off-gas content composition for calculating oxygen uptake rate and carbon dioxide evolution rate of the bioprocess), balances (DEFENDER™ 5000 - D52XW and DEFENDER™ 3000, OHAUS, USA, providing online broth weight and feeding bottle weight of the bioprocess), and house-made sensors that integrated to monitor the bioprocess system (providing extra information about the bioprocess, like off-gas humidity, back pressure of the bioreactor head space, etc.) are now supported by Biofuser. And Biofuser communicates with the instrument with various communication protocols, and now it supports OPC DA/UA, Modbus RTU/TCP, MQTT. Biofuser store the acquired online data from different protocols with a uniform format and does preprocessing for further fusing of data with different time frequency or with different precisions. In addition to these online data acquiring port, Biofuser also supplies user interface for manual input of offline data of the bioprocess.

As shown in Figure 4A, the Biofuser collects data from fermentation process equipment according to the corresponding protocols. The collected data is then processed, converted, and categorized into online data, at-line data, and offline data, based on the data types and values provided in the protocols, and stored in a relational database. Due to differences in equipment data formats and communication protocols between manufacturers, integrating devices from different vendors typically requires additional processing to handle the heterogeneous technical details. For example, the measured data of MAX300-LG, the off-gas mass spectrometry, is encoded as integers with Equation 1 for facilitating storage, and the encoded integer was transmitted through Modbus TCP protocol, and should be decoded back when received.

(1) $$\begin{array}{c} \mathrm{Transmitte}d_{\mathrm{Value}}=\frac{\mathrm{Measure}d_{\mathrm{Value}}-\mathrm{ScaleLo}}{\mathrm{ScaleHi}-\mathrm{ScaleLo}}\times \mathrm{FullScale} \end{array}$$

where $\mathrm{Transmitte}d_{\mathrm{Value}}$ indicates the data transmitted under Modbus TCP. $\mathrm{Transmitte}d_{\mathrm{Value}}$ is the preset minimum detection value, ScaleHi is the preset maximum detection value, and FullScale is the maximum transmission value. Biofuser performs decoding after received the signal and then store it into the database.

Data from different sources must be integrated properly (see Figure 4A, steps 1 and 2). The online data from bioreactor is collected every minute, while the measurement frequency for each channel (one channel corresponding to one bioreactor) off-gas spectrometry is around 20 min. As a result, Biofuser first should interpolate the off-gas data for synchronizing data from the two different sources. For offline data sources, which are always generated with frequency of 2–4 h, Biofuser will first store the data directly, and align with online data through interpolation methods when doing further data analysis.

#### 3.1.2 Data transmission from database to application endpoint

As shown in Figure 4B, Biofuser integrates online data, at-line data, and offline data. At-line raw data, e.g., off-gas data measured by mass spectrometry, is replaced with section-averaged value. Specifically, the at-line data gaps are first filled by averaging adjacent points (step 1). It is then interpolated using Lagrange methods with the online data time points as align points (step 2). Finally, all data streams are Lagrange-interpolated as needed to preserve the online time points (step 3). This process synchronizes the heterogeneous data inputs while prioritizing the online data timing.

Biofuser produces fused data comprising built-in and user-defined parameters (Figure 4A). Built-in parameters include those derived from at-line and online data, such as CER, OUR, and RQ (Equation 2–4). Calculated offline parameters include rates at three levels: process level rates (*R* in the units of amount/time, Equation 5), reactor level rates (*r* in the units of amount/volume/time, Equation 6) and microbe level rates (*q* in the units of amount/cell amount/time, Equation 7), and yield coefficient (*Y*, Equation 8).

(2) $$\begin{array}{c} \mathrm{CER}=\frac{F_{\mathrm{ai}r_{\mathrm{in}}}}{m}*\left(\frac{y_{N2_{\mathrm{in}}}*y_{\mathrm{CO}2_{\mathrm{out}}}}{y_{N2_{\mathrm{out}}}}-y_{\mathrm{CO}2_{\mathrm{in}}}\right)*\frac{600}{22.4} \end{array}$$

(3) $$\begin{array}{c} \mathrm{OUR}=\frac{F_{\mathrm{ai}r_{\mathrm{in}}}}{m}*\left(y_{O2_{\mathrm{in}}}-\frac{y_{N2_{\mathrm{in}}}*y_{O2_{\mathrm{out}}}}{y_{N2_{\mathrm{out}}}}\right)*\frac{600}{22.4} \end{array}$$

(4) $$\begin{array}{c} \mathrm{RQ}=\frac{\mathrm{CER}}{\mathrm{OUR}} \end{array}$$

where $F_{\mathrm{ai}r_{\mathrm{in}}}\;$ is the air inlet flow rate, expressed in ml/min. *m* is the mass of liquid in the fermenter, expressed in kg; $y_{\mathrm{CO}2_{\mathrm{in}}}$, $y_{O2_{\mathrm{in}}}$, $y_{N2_{\mathrm{in}}}$, $y_{\mathrm{CO}2_{\mathrm{out}}}$, $y_{O2_{\mathrm{out}}}$ and $y_{N2_{\mathrm{out}}}$ are the contents of carbon dioxide, oxygen and nitrogen at the inlet and outlet of the bioreactor, respectively, and the unit is %.

(5) $$\begin{array}{c} \frac{d(M_{i})}{dt}=F_{i,\mathrm{in}}\cdot c_{i,\mathrm{in}}-F_{i,\mathrm{ou}t}\cdot c_{i,\mathrm{out}}+R_{i}+T_{i} \end{array}$$

(6) $$\begin{array}{c} r_{i}=\frac{R_{i}}{m_{\mathrm{avg}}} \end{array}$$

(7) $$\begin{array}{c} q_{i}=\frac{R_{i}}{{\left(\mathrm{OD}\cdot m\right)}_{\mathrm{avg}}} \end{array}$$

(8) $$\begin{array}{c} Y_{i/j}=\frac{R_{i}}{R_{j}}=\frac{r_{i}}{r_{j}}=\frac{q_{i}}{q_{j}} \end{array}$$

where $\frac{d(M_{i})}{dt}$ is the cumulative amount of substance *i* in time *t*; $F_{i,\mathrm{in}}\cdot c_{i,\mathrm{in}}$ is the flow rate of the reactor into substance *i*; $F_{i,\mathrm{ou}t}\cdot c_{i,\mathrm{out}}$ is the outflow rate of substance *i* from the reactor; $R_{i}$ is the reaction rate of substance *i* in the reactor; $T_{i}$ is the rate at which *i* is transferred to other terms; $m_{\mathrm{avg}}$ is the mean mass at time *t*; and $(\mathrm{OD}\cdot m{)}_{\mathrm{avg}}$ is the average amount of (OD⋅m) at time *t*.

### 3.2 Device faulty identification

Faulty device inspections are critical for accurate process control or process optimization, as signals obtained from Faulty devices may lead to incorrect decisions. The anomaly detection can be implemented by analyzing process data and applying basic rules. Here we take the off-gas mass spectrometry anomaly detection as an example, as accurate data from off-gas mass spectrometers is crucial for deriving key physiological parameters such as CER, OUR, and RQ value. Biofuser diagnoses off-gas mass spectrometer abnormalities using two approaches: (1) Basic characteristic analysis: CO_2_ content average value outside 0.02–0.05 range indicates equipment malfunction; (2) Comprehensive characteristic analysis: High coefficient of variation (CV > 0.1) for CO_2_ content average value indicates malfunction. Figure 5 shows examples of the basic (Figure 5B) and comprehensive (Figure 5C) methods, identifying time periods where the spectrometer was diagnosed as abnormal.

### 3.3 Bioprocess key feature analysis

Key feature analysis utilizing statistical methods is essential for fermentation process optimization. By identifying the critical process parameters that significantly impact product titer, rate and yield (TRY), key feature analysis can help to reduce process cost and improve process efficiency. Spearman correlation analysis and principal component analysis (PCA) are two important statistical methods and are applied to do key feature analysis based on fermentation process data.

#### 3.3.1 Using spearman correlation analysis to identify critical process factors

We illustrate the utilization of the Spearman correlation analysis (see Figure 6) integrated into Biofuser for identifying critical process factors by presenting a case study involving the correlation between the TRY of product and process factors in the optimization of the riboflavin production process.

Efficiently identifying process key parameters that are significantly correlated with the TRY of product is crucial for bioprocess optimization. Biofuser provides Spearman correlation analysis which enables rapid identification of possible critical factors. Three types of correlation can be found between product TRY and process variables: (1) Positive correlation, (2) Negative correlation, (3) No obvious correlation.

In the Spearman correlation analysis plot (see Figure 6A), significant correlations are clustered in the upper left region, where product titer, product rate, biomass, CER, air flow rate, OUR, agitation speed, base flow rate, and dissolved oxygen are highly correlated. The Spearman correlation coefficient between biomass and OUR is 0.89, indicating a positive correlation between OUR and cell growth. Thus, it is feasible to directly evaluate cell growth online during the fermentation process using the OUR transmitted by Biofuser. Moreover, there is no significant correlation between product yield and the fermentation process.

Balancing carbon utilization in growth and production is a challenging task to achieve high titer, rate, yield (TRY), and scalability (20). For high-value products, the significant correlation between product titer, product rate, and fermentation process data is critical to improving product yield. For low-value but high-demand products, the significant correlation between product yield, product rate, and fermentation process data is vital to reducing production costs. Riboflavin is a low-value product, but the market demand is high. Figure 6B shows the correlation coefficients between riboflavin's TRY and fermentation process data, where fermentation process parameters such as product titer, product rate, CER, air flow rate, OUR, agitation speed, and base flow rate are significantly positively correlated, while fermentation process parameters such as RQ, pH, and acid flow rate are not significantly correlated with product titer and product rate, and DO is significantly negatively correlated with product titer and product rate. Among fermentation process parameters, DO is coupled with air flow rate and agitation speed, and normal growth of Bacillus subtilis is accompanied by organic acid production (21), which is positively correlated with base flow rate and OUR. Therefore, further research on fermentation process optimization for riboflavin needs to focus on CER, OUR, DO, and base flow rate.

#### 3.3.2 Principal component analysis (PCA) for process factor analysis

PCA has been applied to fermentation process optimization, it can help to identify key process parameters that influence product formation (including penicillin, protease, ethanol etc.) during process (22). The complex fermentation time series data associated with fermentation processes can make it difficult to identify these key parameters. To address this challenge, Biofuser uses PCA analysis among different batches by leveraging the dimension-reducing property of PCA. Two different kinds of PCA approaches were applied in Biofuser. The first approach involved flattening all variables into a single row vector, with each process variable at each time point take one column for each batch. The resulting matrix had each row representing a batch and each column representing a variable value at a specific time point. The first two primary components, which contained the most information of each batch, were extracted and used to represent a batch as a point in the score plot in Figure 7A. The second approach involved packing all batches data together into a two-dimensional matrix, with its rows representing variable values at a specific time point and its columns representing individual process variables. The first primary component, which was a linear combination of all process variables, was plotted against batch time points, with each batch represented as a time along curves in the plot. The loadings of each process variables to the primary component are plotted in Figure 7B.

The first PCA method is employed to assist in the rapid identification of outlier batches and aggregated batches. Outlier batches may represent abnormal batches in the biological process optimization, or may represent excellent batches in the optimization process. Aggregated batches, on the other hand, represent relatively stable or typical batches. As shown in Figure 7A, batch two and batch five are outlier batches. Batch two was contaminated during the fermentation process, which prevented normal operation of the Bacillus subtilis fermentation. Batch five experienced limited growth of Bacillus subtilis due to nitrogen deficiency, which also prevented normal fermentation.

The second PCA method is used to quickly locate changes in certain fermentation parameters during the fermentation process and to compare them across different batches. In a normal and stable fermentation, the PC1-Time graph should show a smooth increase or decrease. When a large peak appears (either positive or negative), it indicates that one or more parameters have undergone significant changes. As shown in the plot (f) of Figure 7B, batch five showed significant positive and negative peaks at 55 and 63 h, respectively. According to the loading data in the plot e, it can be tentatively concluded that at 55 h, the dissolved oxygen may have a significant downward trend, while pH, OUR, CER, agitation speed, air flow rate, and base flow rate may have significant upward trends. Similarly, it can be tentatively concluded that at 63 h, the dissolved oxygen may have a significant upward trend, while pH, OUR, CER, agitation speed, air flow rate, and base flow rate may have significant downward trends. Finally, the corresponding data in batch five was located quickly based on the above tentative conclusions, as shown in plot (g), and the conclusions drawn were well validated. Using the same method, differences in biological process parameters that affect different batches can be quickly identified through PCA analysis. As shown in plot (h), there is a significant difference in PC1 direction between batch 2 and batch 5 at 37 h of fermentation. By identifying the biological process parameters that affect this direction in the loading plot [plot (d)], including pH, OUR, CER, agitation speed, air flow rate, base flow rate, and DO, it is speculated that these parameters have significant differences in the batch data, as shown in plot (i), and the conclusions drawn were well validated.

### 3.4 Bioprocess data prediction

Fermentation process is one of the important production technologies in the field of biomanufacturing. The process is complex, nonlinear, highly uncertain, and time-varying. Traditional hard measurement techniques often require destructive sampling and offline testing, causing certain interference and damage to the fermentation process, and have long testing cycles and limited data accuracy (23). In contrast, soft sensing technology based on multivariate statistical analysis and modeling methods can achieve comprehensive real-time monitoring and optimization control of the fermentation process by online monitoring multiple key parameters (24–26). The advantage of soft sensor technology is that it can obtain real-time information on non-measured variables, such as state changes and abnormal behaviors of the fermentation process, which can help in rapid warning and adjustment, thereby improving the production efficiency and product quality of the fermentation process. Therefore, in the field of biomanufacturing, soft sensing technology has become one of the main means for monitoring and controlling the fermentation process. In this part, the Biofuser connected artificial neural network model (ANN) model is given as an example to illustrate the application of Biofuser in the prediction of biological process data.

Biofuser provides a data pipeline for real-time soft sensing, as shown in Figure 8A. Biofuser organizes the data at each time point into a sample feature, uses the feature values as input, predicts the values through a model, and then integrates the predicted values into the time series to achieve real-time soft sensing. The online data obtained from the Bifuser at a specific time, including stirring speed, dissolved oxygen, feeding rate, pH, temperature, fermentation liquid weight, airflow rate, acid pump speed, and inspection pump speed, were merged with the fused values of CER and OUR to form a set of feature values. The biomass at the same time was taken as the observed value and combined with the feature values to form a training sample. Firstly, a training set was constructed using data from four batches (The fermentation process anomalies were manually removed, resulting in the expansion of the database to 13,486 entries.), which was then split into an 8:2 ratio with 8 parts used for model training and 2 parts used for model validation. Next, a six-layer neural network was used as the soft measurement model, and the training results are shown in Figure 8B, indicating that both the training and validation have good loss values. Finally, the trained soft measurement model was incorporated into the data pipeline for biomass soft measurement, as shown in Figure 8C. In the early stage of fermentation (before 34 h), the model's predicted values were close to the actual measured values, showing good prediction performance. However, in the later stages of fermentation, predicted values were 11.0%–19.4% lower than actual measured values. The underlying reason for this phenomenon is that the predicted biomass derived from online data relies on the optical density at 600 nm (OD600), which can effectively approximate the number of living cells during the early fermentation stage and result in a relatively small error in biomass prediction. However, as cells age and die, the OD600 value may still be high, leading to an overestimation of biomass in the late fermentation period.

## 4 Conclusion

In this paper, we have discussed the challenges in optimizing biological processes and how these challenges have been addressed by Biofuser. Specifically, the challenges of data standardization, data management, multiple and complex equipment, and limited data sources have been addressed by Biofuser's comprehensive relational database system, data acquisition and processing technology, and data fusion capability using the Django framework. The importance of Biofuser in optimizing biological processes can be seen in its ability to reduce human error and cost, provide stable and accurate data, and enable the integration of information from multiple sources to gain a more comprehensive understanding of biological processes.

Further research on Biofuser is needed to continue to improve its capabilities. First, research on advanced data acquisition and processing technologies, such as image recognition and natural language processing, can help expand the scope of data collection and improve data quality. Second, research on process analysis methods can provide more accurate and efficient analysis of fermentation processes. Finally, the integration of artificial intelligence can enhance the capability of Biofuser in data analysis, prediction, and decision-making, leading to more efficient and effective optimization of biological processes.

In conclusion, Biofuser has made significant contributions to optimizing biological processes by addressing the challenges of data standardization, data management, and limited data sources. Further research on Biofuser can improve its capability in data acquisition and processing, process analysis, and integration of artificial intelligence, leading to more efficient and effective optimization of biological processes.

## Data Availability

The data that support the findings of this study are available from the corresponding author upon reasonable request.
